# The impact of successful chronic total occlusion percutaneous coronary intervention on long-term clinical outcomes in real world

**DOI:** 10.1186/s12872-021-01976-w

**Published:** 2021-04-15

**Authors:** Xuhe Gong, Li Zhou, Xiaosong Ding, Hui Chen, Hongwei Li

**Affiliations:** 1 Department of Cardiology, Cardiovascular Center, Beijing Friendship Hospital, Capital Medical University, Beijing, 100050 People’s Republic of China; 2 Department of Internal Medicine, Medical Health Center, Beijing Friendship Hospital, Capital Medical University, Beijing, 100050 People’s Republic of China; 3 Beijing Key Laboratory of Metabolic Disorder Related Cardiovascular Disease, Beijing, 100069 People’s Republic of China

**Keywords:** Chronic total occlusions (CTOs), Percutaneous coronary intervention (PCI), Revascularization, Major adverse cardiac and cerebrovascular events

## Abstract

**Background:**

Coronary chronic total occlusions (CTOs) are related to increased risk of adverse clinical outcomes. The optimal treatment strategy for CTO has not been well established. We sought to examine the impact of CTO percutaneous coronary intervention (PCI) on long-term clinical outcome in the real world.

**Methods:**

A total of 592 patients with CTO were enrolled. 29 patients were excluded due to coronary artery bypass grafting (CABG). After exclusion, 563 patients were divided into the no-revascularized group (CTO-NR group, n = 263) and successful revascularized group (CTO-R group, n = 300). The primary endpoint was cardiac death; secondary endpoint was major adverse cardiac and cerebrovascular events (MACCE), a composite of all-cause death, cardiac death, recurrent myocardial infarction, target lesion revascularization, re-hospitalization, heart failure, and stroke.

**Results:**

Percent of Diabetes mellitus (53.2% vs 39.7), Chronic kidney disease (8.7% vs 3.7%), CABG history (7.6% vs 1%), three vessel disease (96.2% vs 90%) and left main coronary artery disease (25.1% vs 13.7%) was significantly higher in the CTO-NR group than in success PCI group (all *P* < 0.05). Moreover, the CTO-NR group has the lower ejection fraction (EF) (0.58 ± 0.11 vs 0.61 ± 0.1, *p* = 0.001) and fraction shortening (FS) (0.31 ± 0.07 vs 0.33 ± 0.07, *p* = 0.002). At a median follow-up of 12 months, CTO revascularization was superior to CTO no-revascularization in terms of cardiac death (adjusted hazard ratio [HR]: 0.27, 95% conference interval [CI] 0.11–0.64). The superiority of CTO revascularization was consistent for MACCE (HR: 0.55, 95% CI 0.35–0.79). At multivariable Cox hazards regression analysis, CTO revascularization remains one of the independent predictors of lower risk of cardiac death and MACCE.

**Conclusions:**

Successful revascularization by PCI may bring more clinical benefits. The presence of low left ventricular ejection fraction (LVEF) and LM-disease was associated with an incidence of cardiac death; CTO revascularization was a protected predictor of cardiac death.

**Graphical abstract:**

Successful revascularization by PCI offered CTO patients more clinical benefits, manifested by lower incidence of cardiac death during follow-up. The presence of LVEF < 0.5 and left main coronary artery disease (LM disease) was associated with an incidence of cardiac death; CTO revascularised was a protected predictor of cardiac death.
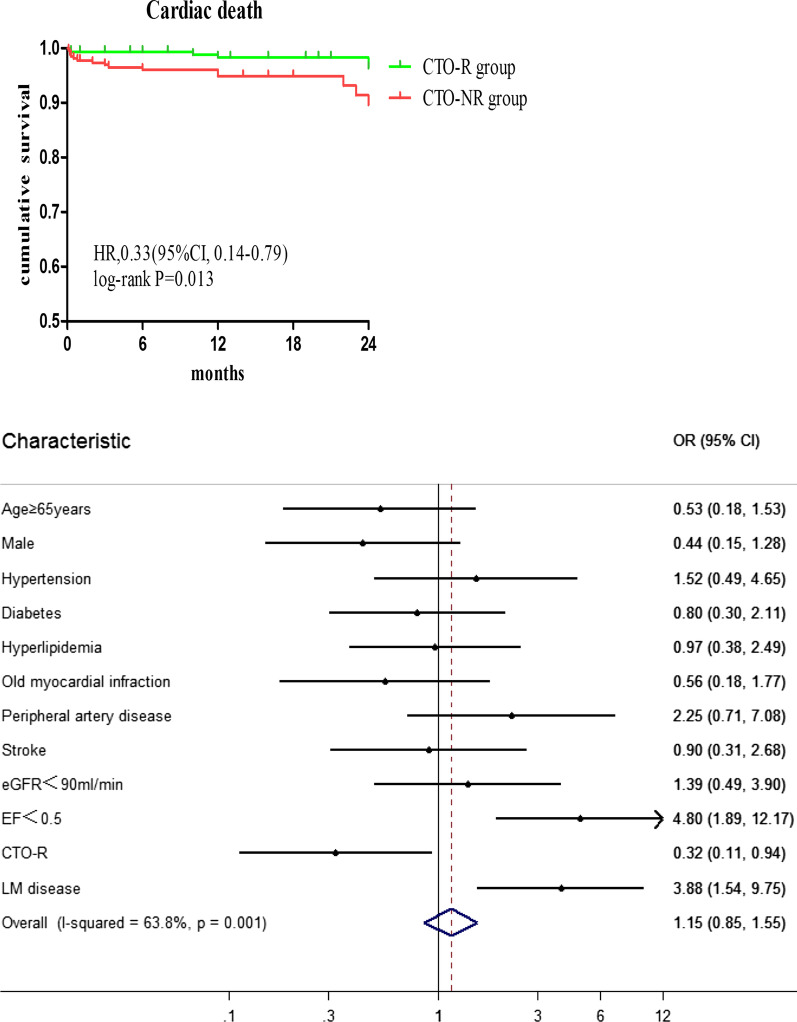

## Background

Coronary chronic total occlusions (CTO) are associated with adverse clinical outcomes. With the development of interventional therapy equipment and technologies, percutaneous coronary intervention (PCI) of CTO is increasingly pursued, and the success rate is getting higher. However, its beneficial effect remains debatable. Some observational studies comparing successful versus failed PCI for CTO have demonstrated that successful PCI was associated with better outcomes, while a few randomized controlled trials (RCTs)’s conclusions did not consistently support the benefit of CTO PCI. Moreover, this benefit is only the improvement of clinical symptoms [1, 2]. Overall, it remains unclear whether CTO revascularization brings long-term clinical benefit. Therefore, we assessed whether successful revascularization offers a clinical benefit in the CTO patients. This study focused on the major adverse cardiac and cerebrovascular events (MACCE), the MACCE includes all-cause death, cardiac death, recurrent myocardial infarction, heart failure, target lesion revascularization, re-hospitalization, and stroke.

## Methods

### Study design and population

**Fig. 1:**
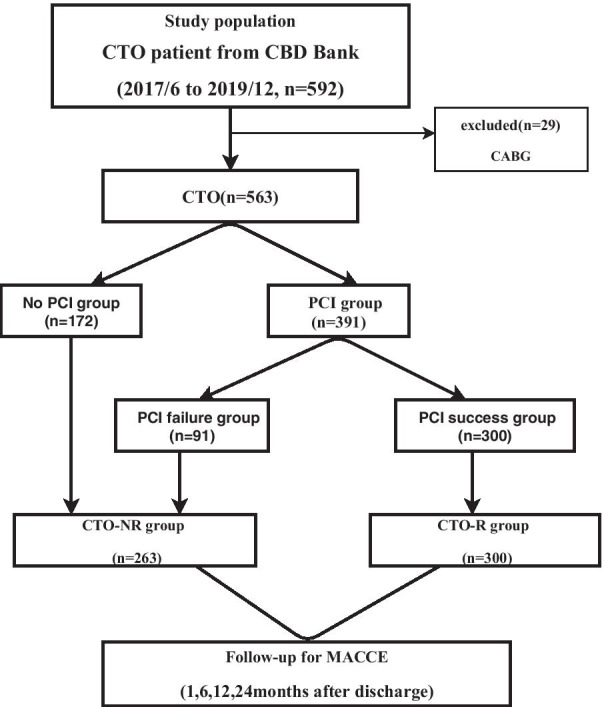
Flow chart of patient enrollment, MACCE: major adverse cardiac and cerebrovascular events, CTO-R: CTO revascularized. CABG: coronary artery bypass grafting

The retrospective study was based on data from the Cardiovascular Center of Beijing Friendship Hospital Data Bank (CBD Bank). The protocol was approved by the ethical committee of Beijing Friendship Hospital. From June 2017 to October 2019, coronary angiography was performed in 6428 consecutively patients at our center, a total of 592 patients (9.2%) with CTO were enrolled in this study. 29 patients were excluded because they received CABG treatment after the CTO diagnosis. Finally, a total of 563 patients were included in the final analysis. Among the 563 patients, 19 cases were diagnosed with stable angina pectoris (SAP), 370 cases had unstable angina pectoris (UAP) and 174 cases had acute myocardial infarction. Patient flow of the study is shown in Fig. 1.

### Definitions of variables and clinical endpoints

The patients’ demographic data, past medical history (hypertension, coronary heart disease, diabetes, hyperlipemia and other diseases), and the conditions of smoking and drinking, were collected from medical records. Body mass index (BMI) was calculated by weight (kilograms) divided by the square of height (meters). The estimated glomerular filtration rate (eGFR) was calculated by standard calculations (GFR based on levels of creatinine [GFR(epi)]) [3].

For the angiogram data, experienced, interventional physicians rescanned the cine angiograms by using standard definitions in order to reduce bias. The definition of CTO was a TIMI flow grade of 0 and an estimated duration of at least 3 months. CTO revascularization (CTO-R) was defined as final residual stenosis less than 20%, with TIMI grade ≥ 2 flow on visual assessment [4]. CTO no-revascularization (CTO-NR) included patients who not PCI and failed PCI. Patients selected for PCI showed symptomatic angina, and/or myocardial viability in the territory of CTO. Moreover, the willingness of patients to undergo PCI treatment was also an influencing factor of the treatment decision. There, the treatment was based on the common decision of doctors and patients. This is highly consistent with clinical practice.

The primary endpoint was cardiac death, defined as previously reported including death from AMI, heart failure, arrhythmia and unexplained sudden death [5]. The secondary endpoint was major adverse cardiac and cerebrovascular events (MACCE), a composite of all-cause death, cardiac death, recurrent myocardial infarction, target lesion revascularization, re-hospitalization, heart failure, and stroke.

All the information was recorded from new hospitalizations or by telephone calls and/or ambulatory visits performed 6 months after PCI and at least one time per year. Clinical follow-up was censored at the date of last follow-up or at 2 years, whichever came first.

### Statistical analysis

Demographic and clinical factors were compared between cohorts of patients depending on the result of CTO revascularization and the different coronary artery CTO lesions. Baseline characteristics were described with mean ± SD (or medians and first and third IQRs) for continuous variables and compared with Student's t test or Mann–Whitney or Wilcoxon tests. Categorical variables were expressed as frequencies and percentages, and were compared by Chisquare or Fisher’s extract statistics.

Survival curves were conducted using Kaplan–Meier estimates and compared with the log-rank test. Cox proportional hazards methods were used to estimate the effect of multiple independent variables on the risk of adverse clinical events in both CTO-R and CTO-NR patients. All factors showing significance in the univariate analysis (*p* < 0.05) were then examined by a multivariate analysis. The results are reported as adjusted hazard ratios (HR) with associated 95% Confidence Intervals (CI). All analyses were 2-tailed and a *P* value < 0.05 was considered statistically significant. All analyses were performed by using SPSS (version 25.0, Chicago, IL, USA) and Metaninf function in Stata 12.0; Kaplan–Meier survival curves were generated with the use of GraphPad Prism software (version 5; GraphPad, Inc, San Diego, CA).

## Results

A total of 563 CTO patients were consecutively enrolled in the present study (Fig. 1). Of these patients, 300 (50.7%) were managed by successful PCI revascularization (CTO-R group) and 263 (44.4%) were not revascularized (CTO-NR group). The CTO-NR group included patients who initial CTO-no PCI (n = 172) and failed CTO-PCI (n = 91). The PCI success was around 77% (300 success/391 attempts). In regard to periprocedural complications, coronary artery perforation occurred in 2 patients, coronary artery dissection occurred in 1 patient, coronary thrombotic event occurred in 1 patient, and myocardial infarction occurred in 1 patient during perioperative period.

### Baseline characteristics

The baseline clinical characteristics of different coronary artery CTO lesions are reported in Table 1. The CTO lesions were located in LAD: 163 (27.5%), LCX: 148 (25%), RCA: 218 (36.5%) and ≥ 2 vessel: 61 (27.5%). Patients with CTO in ≥ 2 vessel had higher incidence of previous history of CABG and choosing CABG treatment compared to those with CTO lesions in LAD, LCX or RCA. Moreover, the proportion of successful PCI in LAD group was the highest.

**Table 1:** Baseline characteristics of the study population grouped by the different coronary artery CTO lesions

Variable	LAD (n = 163)	LCX (n = 148)	RCA (n = 216)	≥ 2 vessel (n = 65)	*P*
Age (years)	64.74 ± 9.98	64.37 ± 10.23	64.66 ± 10.7	64.25 ± 11.67	0.982
Male	136 (83.4)	119 (80.4)	172 (79.6)	52 (80)	0.811
Days	7 (5,9)	6 (5,8)	7 (5,9)	7 (5,10.5)	0.291
BMI (kg/m^2^)	25.83 ± 3.3	26.25 ± 3.33	25.94 ± 3.37	26.16 ± 4.16	0.717
AC (cm)	93.14 ± 9.7	94.06 ± 10.08	93.32 ± 9.44	93.29 ± 12.11	0.868
Smoke	102 (62.6)	94 (63.5)	153 (70.8)	42 (64.6)	0.312
Drink	31 (19)	20 (13.5)	54 (25)	14 (21.5)	0.059
HT	113 (69.3)	112 (75.7)	162 (75)	47 (72.3)	0.553
DM	78 (47.9)	72 (48.6)	87 (40.3)	37 (56.9)	0.086
Hyperlipemia	91 (55.8)	92 (62.2)	118 (54.6)	35 (53.8)	0.486
CHD	94 (57.7)	88 (59.5)	140 (64.8)	40 (61.5)	0.525
OMI	32 (19.6)	29 (19.6)	56 (25.9)	16 (24.6)	0.375
HF	2 (1.2)	1 (0.7)	3 (1.4)	2 (3.1)	0.577
CKD	8 (4.9)	10 (6.8)	13 (6)	3 (4.6)	0.879
PAD	9 (5.5)	13 (8.8)	20 (9.3)	4 (6.2)	0.519
Stroke	36 (22.1)	38 (25.7)	42 (19.4)	12 (18.5)	0.486
History of PCI	49 (30.1)	48 (32.4)	64 (29.6)	19 (29.2)	0.94
History of CABG	3 (1.8)	2 (1.4)	8 (3.7)	10 (15.4)	**< 0.001**
ISR	15 (9.2)	5 (3.4)	17 (7.9)	2 (3.1)	0.104
PCI open	107 (68.2)	58 (40.6)	111 (53.9)	24 (42.1)	**< 0.001**
CABG	6 (3.7)	5 (3.4)	10 (4.6)	8 (12.3)	0.03

Bold values indicate that the difference is statistically significant (*p* < 0.05)

Data are presented as absolute numbers and percentages (for categorical variables) or mean value ± SD (for continuous variables) unless otherwise specified. BMI, body mass index; AC, Abdominal circumference; HT, hypertension; DM, diabetes mellitus; HF, heart failure; CHD, coronary heart disease; OMI, old myocardial infarction; CKD, Chronic kidney disease; PAD, Peripheral arterial disease; CABG, coronary artery bypass grafting; CTO, coronary chronic total occlusion; PCI, percutaneous coronary intervention; ISR, In stent restenosis

Table 2 shows the differences of clinical characteristics with CTO-R group and CTO-NR group. Compared to CTO revascularized patients, those not revascularized had a higher prevalence of diabetes, chronic kidney disease, prior CABG, three-vessel disease and LM disease, moreover, these patients were significantly older (67 ± 11 vs 63 ± 10 years; *p* = 0.000), with lower mean ejection fraction (0.58 ± 0.11 vs 0.61 ± 0.1; *p* = 0.001) and fraction shortening (0.31 ± 0.07 vs 0.33 ± 0.07; *p* = 0.002), the level of CHOL and LDL-C were also lower. Whereas the peak concentration of NT-proBNP was higher (487 vs 278, *p* = 0.001) in the CTO-NR group. For the other variables, there was no significant difference between the two groups.

**Table 2:** Baseline characteristics of patients stratified for CTO lesion Revascularized or Not

Variable	CTO-R group (n = 300)	CTO-NR group (n = 263)	*P*
Age	63 ± 10	67 ± 11	**< 0.001**
Male	248 (82.7)	208 (79.1)	0.28
Days	7 (5,9)	6 (5,8)	0.721
BMI (kg/m^2^)	26.19 ± 3.59	25.85 ± 3.2	0.242
AC (cm)	93.36 ± 9.96	93.67 ± 9.77	0.721
Smoke	200 (66.7)	171 (65)	0.681
Drink	63 (21)	50 (19)	0.557
HT	220 (73.3)	193 (73.4)	0.989
DM	119 (39.7)	140 (53.2)	0.001
Hyperlipemia	162 (54)	157 (59.7)	0.174
CHD	177 (59)	167 (63.5)	0.275
OMI	58 (19.3)	68 (25.9)	0.064
HF	4 (1.3)	4 (1.5)	0.851
CKD	11 (3.7)	23 (8.7)	0.012
PAD	21 (7)	21 (8)	0.657
Stroke	63 (21)	58 (22.1)	0.761
History of PCI	88 (29.3)	88 (33.5)	0.292
History of CABG	3 (1)	20 (7.6)	**< 0.001**
CHOL (mmol/L)	4.13 ± 1.09	3.9 ± 1.01	0.013
LDL-C (mmol/L)	2.34 ± 0.76	2.2 ± 0.73	0.036
eGFR (ml/min)	86.24 ± 20.57	80.15 ± 23.37	0.001
NT-proBNPmax (Pg/ml)	278 (81,1034.75)	487 (144,2223)	0.001
LVEDD	5.23 ± 0.64	5.27 ± 0.59	0.43
LVEF	0.61 ± 0.1	0.58 ± 0.11	0.001
FS	0.33 ± 0.07	0.31 ± 0.07	0.002
ISR	25 (8.3)	12 (4.6)	0.072
three vessel disease	270 (90)	253 (96.2)	0.004
LM disease	41 (13.7)	66 (25.1)	0.001

Bold values indicate that the difference is statistically significant (*p* < 0.05)

Values are n (%), mean ± SD or median with interquartile range. CHOL, cholesterol; LDL-c, low-density lipoprotein cholesterol; NT-proBNP, N-terminal pro-brain natriuretic peptide, eGFR, estimated glomerular filtration rate; LVEDD, left ventricular end-diastolic dimension, LVEF, left ventricular ejection fraction; FS, fraction shortening, LM disease, Left main disease

**Fig. 2:**
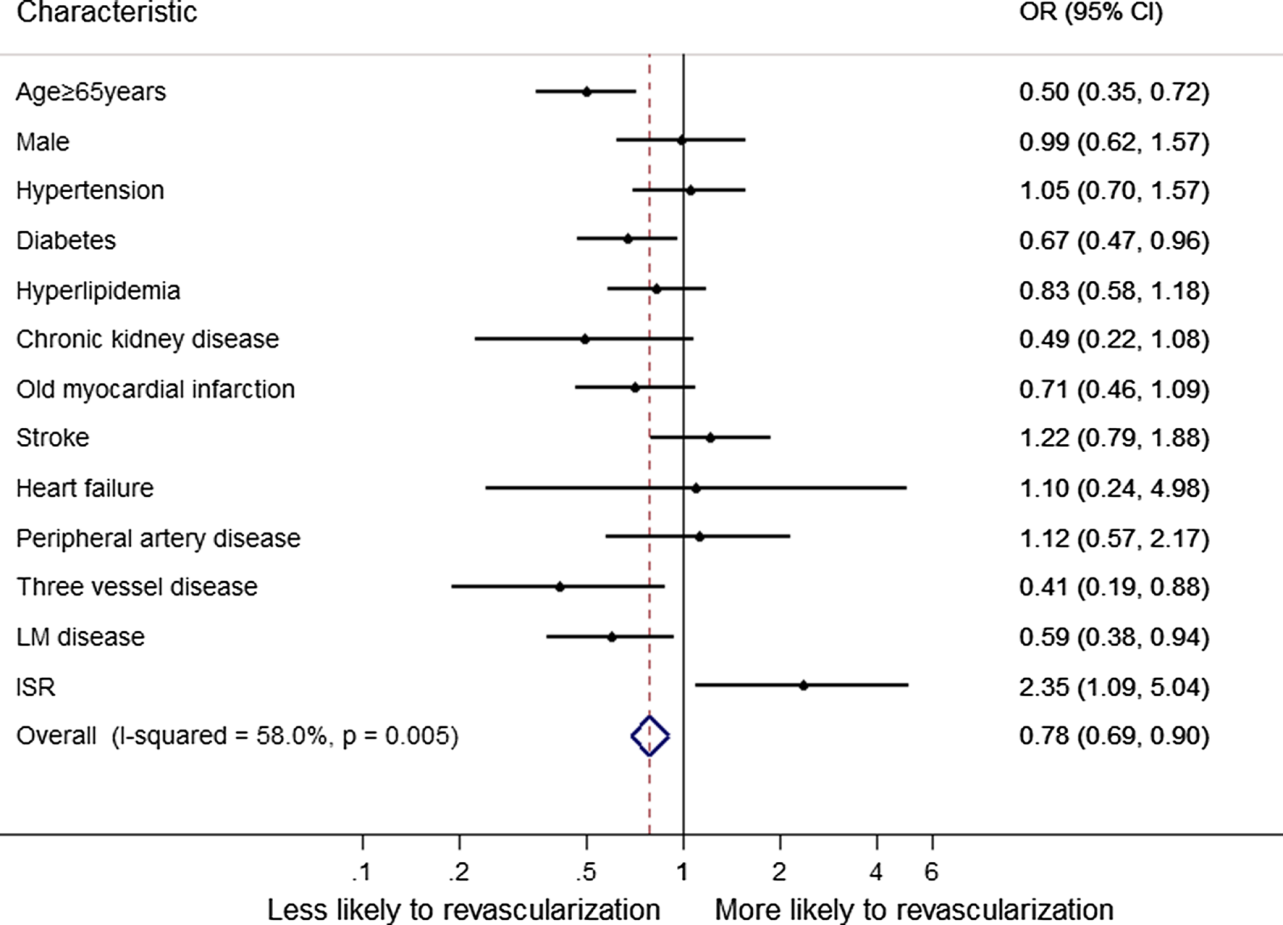
Factors associated with CTO revascularization in multivariable analysis. Variables associated with CTO revascularization are shown along the vertical axis. The strength of effect is shown along the horizontal axis with the vertical line demarcating an odds ratio (OR) of 1 (i.e., no association); estimates to the right (i.e., > 1) are associated with a greater likelihood of CTO revascularization, whereas those to the left (i.e., < 1) indicate a reduced likelihood of CTO revascularization. Each dot represents the point estimate of the effect of that variable in the model, whereas the line shows the 95% confidence interval (CI)

Significant correlates of CTO revascularized in multivariable analysis are shown in Fig. 2. Compared with CTO-R group, no revascularized patients were more likely to be older (age ≥ 65 years), and the proportion of diabetes, three-vessel disease and LM disease is higher. Also, the CTO-R group was more likely to be in-stent (IS) CTO.

### Clinical follow-up

**Fig. 3:**
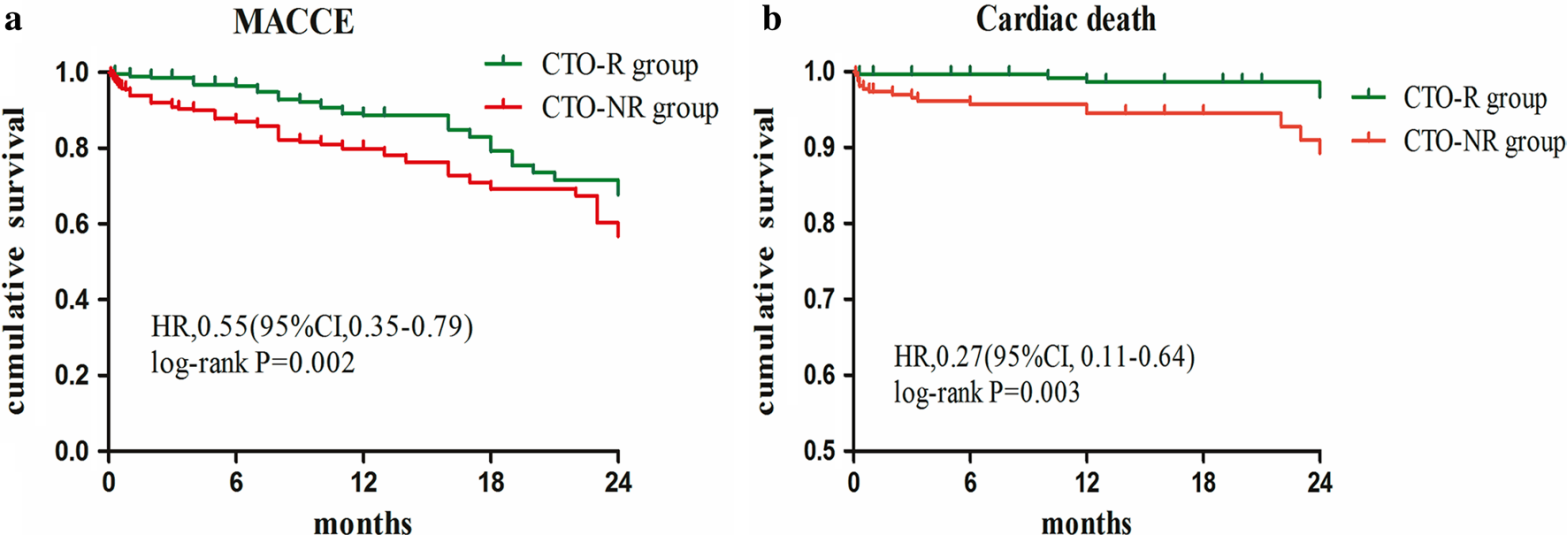
Kaplan–Meier analysis of MACCE (secondary endpoint, panel **a**) and cardiac death (panel **b**) for overall patients stratified for CTO revascularised (R: green continuous line) and not revascularised (NR: red dotted line)

Up to 2-year follow-up (median 1 years, IQR 6–12 months), compared with CTO-R patients, those with CTO-NR had significantly higher rate of cardiac death (6.1% vs 1.3%; P_log-rank_ = 0.003) and of MACCE (22.1% vs 12%; P_log-rank_ = 0.002) (Fig. 3a, b respectively). Re-hospitalization tended to occur more frequently in CTO-NR patients than in CTO-R patients (17.5% vs 11.3%, *p* = 0.037). Moreover, no revascularized CTO patients suffered more often from target vessel revascularization (2.3% vs 0.3%, *p* = 0.037), and have therefore a higher MACCE rate (*p* = 0.001). Other clinical events are reported in Table 3.

**Table 3:** Comparison of clinical outcome between CTO-R group and CTO-NR group

Event, n (%)	CTO-R group (n = 300)	CTO-NR group (n = 263)	*P*
MACCE	36 (12)	58 (22.1)	0.001
All-cause death	7 (2.3)	17 (6.5)	0.016
Cardiac death	4 (1.3)	16 (6.1)	0.002
Re-hospitalization	34 (11.3)	46 (17.5)	0.037
Heart failure	8 (2.7)	4 (1.5)	0.348
Target vessel revascularization	1 (0.3)	6 (2.3)	0.037
Recurrent myocardial infarction	6 (2)	5 (1.9)	0.933
Stroke	1 (0.3)	3 (1.1)	0.255

MACCE: major adverse cardiac and cerebrovascular events, a composite of all-cause death, cardiac death, recurrent myocardial infarction, target vessel revascularization, re-hospitalization, heart failure, and stroke

### Predictors of survival

Considering all CTO patients, a multivariable Cox regression analysis (Table 4) was used to identify clinical and angiography independent predictors of cardiac death and MACCE. For MACCE, the final multivariable model included LVEF < 0.5 and CTO-R. For cardiac death, the final multivariable model included LVEF < 0.5, CTO-R and LM disease.

**Table 4:** Multivariate Cox regression analysis in the overall CTO patients

	Predictor variable	HR (95% CI)	*P*
MACCE	Age ≥ 65 years	0.92 (0.58–1.465)	0.729
	Male	0.663 (0.398–1.106)	0.116
	HT	0.954 (0.594–1.532)	0.845
	DM	0.822 (0.537–1.26)	0.369
	HP	0.89 (0.588–1.346)	0.581
	OMI	0.85 (0.508–1.422)	0.536
	PAD	1.317 (0.674–2.575)	0.42
	Stroke	1.028 (0.632–1.672)	0.912
	eGFR < 90	0.999 (0.639–1.561)	0.996
	LVEF **< **0.5	2.121 (1.304–3.452)	0.002
	CTO-R	0.541 (0.353–0.83)	0.005
	LM disease	1.211 (0.734–2.001)	0.454
Cardiac death	Age ≥ 65 years	0.501 (0.172–1.459)	0.205
	Male	0.437 (0.148–1.288)	0.133
	HT	1.548 (0.501–4.778)	0.447
	DM	0.781 (0.295–2.065)	0.618
	HP	0.948 (0.365–2.461)	0.913
	OMI	0.548 (0.171–1.75)	0.31
	PAD	2.332 (0.739–7.364)	0.149
	Stroke	0.925 (0.31–2.758)	0.889
	eGFR < 90	1.369 (0.485–3.861)	0.553
	LVEF **< **0.5	4.804 (1.895–12.177)	0.001
	CTO-R	0.239 (0.076–0.751)	0.014
	LM disease	3.884 (1.544–9.771)	0.004

**Fig. 4:**
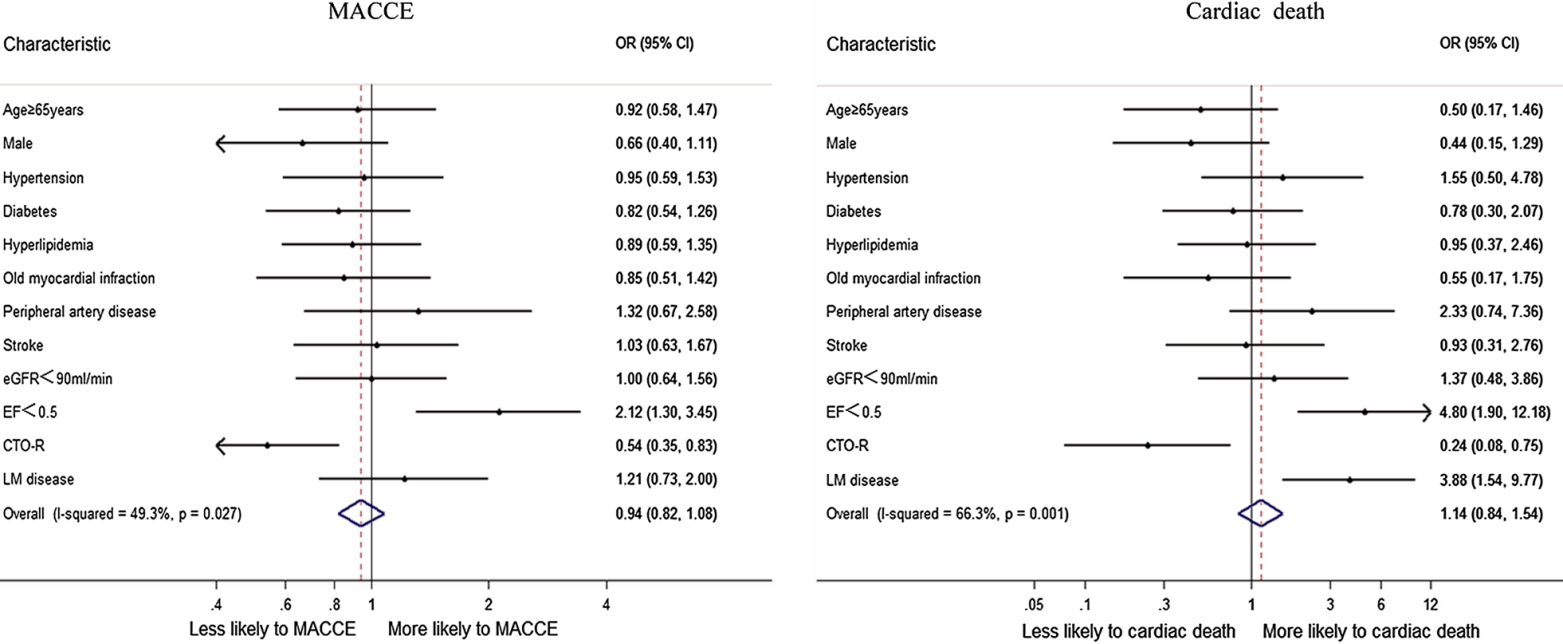
Predictors of cardiac death and MACCE in all CTO patients. CTO-R was a protected predictor of cardiac death and MACCE. Additionally, LVEF < 0.5 and LM disease predicted a worse probability for cardiac death

By multivariate analysis (Fig. 4), CTO-R was a protected predictor of cardiac death (HR: 0.239, 95% CI 0.076–0.751) and MACCE (HR: 0.541, 95% CI 0.353–0.83). Additionally, lower LVEF (LVEF < 0.5, HR: 4.804, 95% CI 1.895–12.177) and LM disease (HR: 3.884, 95% CI 1.544–9.771) predicted a worse probability for cardiac death.

### Effects of different CTO lesion vessels (LAD, LCX and RCA) on MACCE

**Fig. 5:**
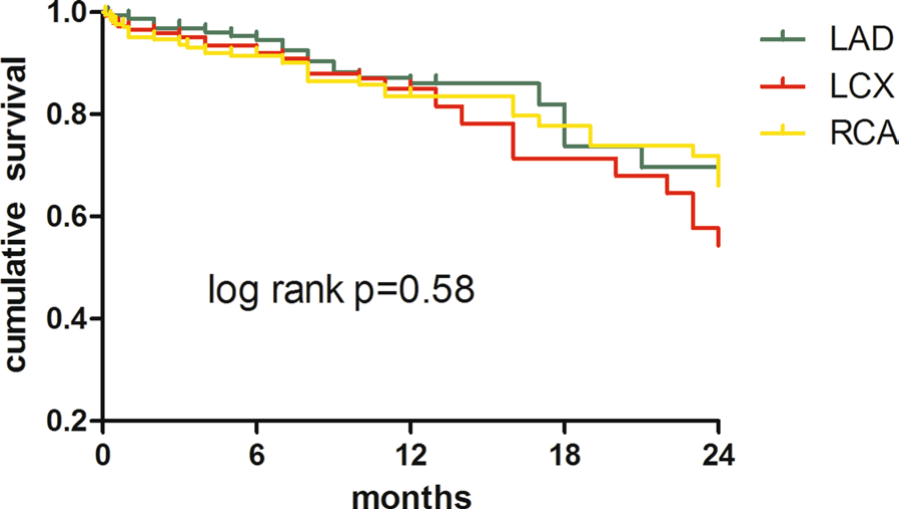
Comparison of Kaplan meire survival curves of different CTO target vessels revascularization

In Fig. 5, the effect of different CTO vessels (LAD, LCX and RCA) revascularization on MACCE was studied, there was no difference in the effect of different CTO lesion vessels revascularization on MACCE (*p* = 0.58). Although there was no statistical difference, the cumulative survival rate of LCX-CTO revascularization is still the lowest.

## Discussion

The present study showed patients with CTO not revascularised by PCI had worse outcomes compared with those with CTO revascularised, with higher incidence of cardiac death and MACCE. The presence of low-LVEF, LM-disease was associated with an incidence of cardiac death. CTO revascularised was a protected predictor of cardiac death.

In our study population, the proportion of older (age > 65 years), diabetes, LM disease and three-vessel disease was higher in the no-revascularised CTO patients. This suggests that CTO with the above characteristics is more difficult to open. More importantly, previous studies have shown that age is an independent risk factor for cardiovascular mortality [6]. Elderly patients with decreased body function are more likely to have hypertension and diabetes, which promote atherosclerosis. In addition, the treatment strategy of CTO will also take into account the age factor; older patients tend to choose conservative treatment rather than PCI intervention. Meanwhile, there were more patients with in-stent restenosis (ISR) CTO in the revascularised group, which is also consistent with clinical practice; the ISR-CTO is easier to open because of the contour of the stent.

Besides, assessing the CTO patient’s heart function is critical to judging the prognosis. Serum NT-proBNP is an important biomarker in our clinical practice. Abnormal elevation of NT-proBNP can accurately reflect the degree of heart failure [7], and its combination with LVEF can comprehensively reflect the state of cardiac function. In our study, the peak of NT-proBNP was higher and the LVEF was lower in the no-revascularised CTO patients. Moreover, low-LVEF was a harmful predictor of cardiac death. We found that the presence of low-LVEF (< 0.5) was associated with an incidence of cardiac death at least 4 times higher than those with LVEF ≥ 0.5. Overall, cardiac dysfunction is more unfavorable to the clinical prognosis of CTO patients, which can significantly increase the incidence of major adverse cardiovascular events.

CTO are a common clinical finding among patients undergoing coronary angiography, approximately 1 in 4 patients with obstructive coronary artery disease on coronary angiography had CTOs [8]. CTO have been referred to as the “final frontier” in interventional cardiology, which are complex and difficult to open [9]. With improvement in equipment and techniques, high success rates can be achieved at experienced centers; PCI for CTOs has been rapidly evolving during recent years. The PCI success was around 77% in our study, which is lower than the classical 90% of the majority of high volume CTO centers. This may be explained by the fact that the patients were too old and have a high proportion of renal insufficiency, and they cannot tolerate long-term surgical operations. Moreover, we compared the success rates of different CTO lesion locations. In regard to success rates, LCX showed lower procedural success rates (40.6%) followed by LAD (68.2%), RCA (53.9%) and ≥ 2 vessel (42.1%). There were significant differences in the success rate of different CTO vessels.

The success rate of LAD-CTO is the highest, the LAD coronary artery supplies a major portion of the left ventricle, its diagonal branches perfuse the entire anterior wall, and its septal branches supply the anterior 2/3 of the septum, especially a proximal LAD-CTO will affect the entire anterior and anteroseptal wall from base to apex. Typically such a lesion, if not revascularized, will compromise overall LV systolic function and reduces the overall LVEF to at least 35–40%, which will lead to hypotension and heart failure [10]. Therefore, clinicians are more willing to try to open LAD-CTO. This may be one of the reasons for the high success rate of LAD-CTO. Nevertheless, the success rate of CTO with more than 2 vessels was low, and the probability of choosing CABG treatment was higher (*p* = 0.03). This result was also consistent with the clinical practice; clinical guidelines recommend CABG as the first choice for multi-vessel disease revascularization. Many studies have suggested a long-term survival advantage for CABG compared with PCI in patients with multi-vessel disease [11]. Moreover, the success rate of LCX-CTO was the lowest; many studies have confirmed that the successful opening of LCX-CTO has not seen obvious clinical benefits [12], and LCX-CTO was more difficult to open, one of the diagnostic criteria of the PROGRESS-CTO score was LCX-CTO [13].

Although successful revascularization of CTO was significantly associated with the decrease of MACCE, in the CTO-R group, different target vessel lesions (LAD, LCX and RCA) revascularization did not affect MACCE. It can be seen from Fig. 5 that successfully opened the LCX-CTO has the least effect on prognosis and the lowest cumulative survival rate. This confirmed the limited clinical significance of opening the LCX-CTO.

Our result showed that successful CTO PCI is associated with a statistically significant improvement in cardiac death and MACCE, successful revascularization of CTO was a protected predictor of cardiac death (HR: 0.239, 95% CI 0.076–0.751) and MACCE (HR: 0.541, 95% CI 0.353–0.83). It may be that the successful opening of CTO can improve cardiac function and ultimately improve the clinical outcome. A previous meta-analysis of 34 studies with 2735 patients on the impact of CTO PCI on LV function was performed in 2018 by Michael Megaly et al. and showed a statistically significant increase in LVEF (3.8%, 95% CI 3.0–4.7, *P* < 0.0001) as compared with baseline [14]. Additionally, in our study, low-LVEF (< 0.5) and LM disease predicted a worse probability for cardiac death. In summary, the predictive risk factors of cardiac death in CTO patients include vascular not revascularised, LM lesions and low-LVEF.

## Limitations

The following limitations were present in this study. (1) This study was a retrospective cohort study. The evidence grade is lower than that of a randomized controlled trial. (2) The signs of a viable myocardium were not all evaluated in our study. (3) Although we used multivariate Cox regression analysis to adjust for differences in baseline characteristics, there may still be unknown confounding factors. Therefore, the research results should be reasonably interpreted. (4) The symptom improvement is one of the benefits of CTO recanalization; the symptom was not assessed in our study.

## Conclusions

In summary, compared with CTO not revascularised, successful revascularization offered patients more clinical benefits, manifested by the lower incidence of cardiac death and MACCE during follow-up. Moreover, the presence of LVEF < 0.5 and LM-disease was associated with an incidence of cardiac death; CTO revascularization was a protected predictor of cardiac death. Further RCTs are needed to investigate the role of PCI for management of patients with CTO.

## Data Availability

The data and materials can be found from the first author and corresponding author.

## Abbreviations

CTO: Chronic total occlusion
PCI: Percutaneous transluminal coronary intervention
ISR: In-stent restenosis
MACCE: Major adverse cardiac and cerebrovascular events
MI: Myocardial infarction
TVR: Target vessel revascularization
LAD: Left anterior descending coronary artery
LCX: Left circumflex artery
RCA: Right coronary artery
CHD: Coronary heart disease
CKD: Chronic kidney disease
LVEF: Left ventricular ejection fraction
BMI: Body mass index
CHOL: Cholesterol
LDL-C: Low-density lipoproteine-cholesterol
HR: Hazard ratio
CI: Conference interval
RCTs: Randomized controlled trials
